# The Hospitals, &c., of New York

**Published:** 1859-11

**Authors:** 


### EDITORIAL DEPARTMENT.

### *The Hospitals, etc., of New York.*

The New York State Emigrants' Hospital and Refuge, Ward's Island.—This institution, which is under the care of the Commissioners of Emigration, is situated on Ward's Island, in the East River, opposite 106th Street. It is, as the name indicates, a Refuge and Hospital for the sick and destitute emigrant on arriving at our port. The Commission was organized about the year 1848, since which time many thousand destitute sick have been cared for annually. The institution is supported by the emigrants themselves, in the following way:—Each one pays the sum of two dollars on entering the port, which entitles him to the protection of the Commissioners until he has been in the country for five years, after which period the obligation ceases.

The Island, though some distance from the center of the City, is quite accessible by the Second and Third Avenue cars, which run every few minutes to within a short distance of the ferry, where boats are always in readiness to carry over visitors. A more agreeable but not so convenient a route is by a steamboat which leaves the foot of Grand Street every day at 12 o'clock, touching at Blackwell's Island.

The medical and surgical departments of the Hospital are each under the charge of a chief, who has a number of younger medical men as assistants. The departments are then subdivided and distributed in small buildings, entirely distinct from each other, scattered over the island, each containing two or more separate wards, with the necessary rooms for nurses, &c. In one of these buildings there is an ample amphitheater in which operations are publicly performed and clinical instruction given. The Physician-in-Chief, Dr. George Ford, is required to reside on the island, as are the assistant physicians and surgeons. Dr. J. M. Carnochan, Professor of Surgery in the New York Medical College, is Surgeon-in-Chief. He visits the island several times a week, and holds a weekly clinic on Saturdays. The clinical material here is ample; and many important operations are yearly performed before the class, among which have been ligatures of large vessels, resections of the bones and joints, amputations, &c., as well as the more delicate surgical operations, as those upon the eye and about the face. Many of Dr. Carnochan's most important operations, and those with which his name has become so honorably connected, have been performed on this island. Students also have an opportunity of following fatal cases to the dead house, and witnessing *post-mortem* examinations, and examining pathological specimens. The cases on the island are most of them acute; and by a reference to the following table, showing the principal diseases treated in 1856–57, the reader will see that they are such as occur in the every-day practice of the physician:

| | | |
|---|---|---|
| Of Abscess there occurred | 90 | cases. |
| " Uterine diseases | 120 | " |
| " Diseases of the Eye | 550 | " |
| " Diarrhœa | 200 | " |
| " Dysentery | 110 | " |
| " Epilepsy | 40 | " |
| " Erysipelas | 40 | " |
| " Fevers of various forms | 450 | " |
| " Phthisis | 20 | " |
| " Venereal Disease | 500 | " |
| " Hernia | 20 | " |
| " Injuries | 90 | " |
| " Bright's Disease | 20 | " |
| " Rheumatism | 200 | " |
| " Ulcers | 250 | " |
| " Elephantiasis | 3 | " |
| " Cutaneous Diseases | 40 | " |
| " Pneumonia | 300 | " |

It is not attempted in this table to give a minute statistical account of the diseases treated on the island, but to give an idea of the amount and quality of the material for clinical instruction. The Hospital is free to medical students, who have only to go and take advantage of the opportunities presented to them; and every year several graduates are appointed as assistants. The opportunities for these gentlemen, it will be seen, cannot be surpassed, and nowhere could one prepare himself better for the actual practice of his profession.

The medical department is divided into the male, female, lying-in, and refuge; the latter including the nursery. This is under the charge of the Physician-in-Chief; and in the male and female departments, the student has the opportunity of studying all forms of disease, especially fevers and diseases of the respiratory organs. In the Refuge there are many chronic cases, women awaiting childbirth, and children up to the age of twelve years. This department affords a fine field for the observation of diseases incident to advanced pregnancy, infancy and childhood, which the young practitioner knows he is frequently called upon to treat, and which are often the most puzzling and annoying. There is also an insane department, under the direction of the Physician-in-Chief. Thus, the student has an opportunity of studying the diseases of the mind, as well as those of the body.

The reports show that there have been, for a number of years, an average of four hundred and fifty births, and nine thousand patients treated annually, including diseases common to all ages, with a majority of those incident to adult life.

The surgical department, male and female, has a syphilitic, ophthalmic, and a division of general surgery. This is under the charge of Dr. Carnochan, and affords fine opportunities for the study of surgical diseases. The surgical clinics of this distinguished teacher cannot fail to be highly instructive, presenting as he does the latest and soundest views on surgical practice.

Having thus sketched the clinical advantages which are at the command of the student at Ward's Island, the reader will see that they form an important part of the great clinical resources of the Metropolis; and being one only of the numerous places where graduates can pass a time as resident assistants, the appreciation of these resources by the profession at large cannot fail to make New York the great center for medical instruction.

St. Luke's Hospital.—There has recently been added to the corps of medical institutions of New York city, one which has already gained an eminent position, not only from its usefulness, but especially from the peculiar interest which attaches to it as a novel experiment in this country. I refer to St. Luke's Hospital. In this institution, religion holds a prominent and essential position, and its ministrations combine with those of medical science, for the relief of diseased humanity.

"Corpus sanare, animam salvare."

Such is its motto, and such the spirit upon which it is conducted.

The hospital was founded and is supported by the Protestant Episcopalians of New York. The project was conceived and carried out by the Rev. Dr. Muhlenberg, the venerable pastor of the "Church of the Holy Communion," to whose devoted zeal, as its religious and business head, its success and continued usefulness are mainly due.

The great distinguishing feature, however, of this institution is the fact, that the wards and their inmates are under the immediate charge of a Christian sisterhood of ladies—intelligent, refined and educated women, who have given their lives to this noble charity, and to whose untiring and enlightened efforts at the bedside of the sick and dying, the peculiar beauty and elevated character of this institution are to be ascribed.

Much might be said of the blessings which accrue to the patient from the constant presence of Christian example, and the timely ministration of religious exhortation; but such is not the object of the present article. It is simply of its qualities in a medical point of view, that I am now to speak.

The Hospital is situated upon 5th Avenue, 54th and 55th Streets. It stands back from the street, between which and its imposing front stretches a broad lawn. The edifice is of brick, with brown-stone trimmings, and is built after the Norman style of architecture. At the central portion, and flanking the entrance on either side, rise two lofty square towers, inclosing between them the chapel, surmounted by a stone cross; while on the right and left again stand the hospital wings, three stories in height, the whole presenting a front of 280 feet.

The internal arrangement is very simple, and, at the same time, most admirably adapted to the purposes for which it was intended. Ventilation is most perfect, there being but a single range of wards between the external walls, and these bounded again by broad and airy corridors; while to avoid the slightest possibility of contamination, a large revolving fan is connected with a steam engine in an adjoining building, and so placed as to drive a current of fresh air directly through a large flue into the Hospital, thus changing in a few moments the entire atmosphere of the house. The wards are 100 feet in length by 30 feet in breadth; and along either side are ranged the white-curtained beds, each furnished with a table and a small carpet, the whole presenting the most unsullied cleanliness and order. The wards terminate in the central chapel; and here every night such of the patients as are able assemble at evening prayer, while the acoustic properties of the building are such as to render the voice of the speaker audible to the farthest extremity of the wings. There are also smaller wards, containing but two or three beds each, for such cases as may, for any reason, demand seclusion.

On the third floor is a light and convenient operating-room; and in the basement a well-stocked drug store, with a full complement of surgical instruments and appliances. The building will accommodate two hundred patients.

The medical charge of the Hospital is committed to a Board consisting of four consulting and four attending physicians, four consulting and four attending surgeons, a pathological chemist, an admitting physician, and a resident. One physician and one surgeon are in constant daily attendance. Each ward is under the direct superintendence of a "Sister," who sees that each prescription is administered, and each order carried out as directed by those in attendance, while the menial offices are performed by nurses, male or female as the case may be, under her supervision.

None can appreciate so well as medical men, the immense advantages arising out of such a system. The perfect accuracy and reliability which are the result, cannot fail to render the treatment of medical or surgical disease in the highest degree satisfactory to the practitioner, and beneficial to the patient; while the good accruing to science, from observations made under such circumstances, can hardly be overrated.

The economy of the Hospital is so arranged as to be adapted to the wants, not alone of the destitute and those of moderate means, but so far as may be, of every class in society. A bed in one of the general wards may be had for a few dollars per week by those who are able to pay; while those who are too poor for this are gladly accommodated gratuitously. But this is not all. There are private rooms, pleasantly and comfortably furnished, with bath and other conveniences attached, for the accommodation of strangers taken sick in the city, and others who desire the comfort and security of hospital treatment, and yet wish to enjoy the luxury and attention to which their circumstances entitle them. In fact, throughout the entire arrangement and conduct of the Hospital, the grand idea is to make it a home for those who enter its doors—that every patient may feel that he is surrounded by friends, and that from the moment he sets foot within the walls of St. Luke's he is no longer a stranger, but a member of a family, watched over, not by hirelings, but by those whose greatest pleasure is to relieve his sufferings, and to restore him to health their highest satisfaction.

A moment's reflection will show how great a boon is such an asylum to a city like New York, where every year hundreds of poor but respectable artisans, of both sexes, suffer and die for the want of decent care when sick; a class of the community whose labor brings them just enough to support them in health, but who, when that fails, are unable to purchase good attendance, and who yet shrink, with an ill-defined but natural dread, from the associations with which the wards of a pauper-hospital will inevitably surround them. Although, as I have said, freely open to all, it is more especially to this class that St. Luke's would stretch forth a sympathizing and a helping hand.

Founded and sustained by Episcopalians, St. Luke's Hospital is yet not in the slightest degree a sectarian institution. Every denomination is alike welcome, and the same liberal Christian spirit prompts the treatment of every patient under its roof.

As yet the wards are not thrown open to students of the profession; but from what has been said, it will readily be seen how superior would be its advantages for clinical instruction, should it at any time be thought advisable thus to extend its sphere of usefulness.

The New York Preparatory School of Medicine.—We have received the circular of this institution for the coming season, when it goes into operation, for the first time, as an incorporated school. The faculty is composed of eight members, seven of them occupying the departments usually allotted to the different members of the faculties of American medical colleges, and the eighth holding the chair of legal medicine. The faculty is authorized, by the act of incorporation, to confer the degree of Bachelor of Medicine, upon the candidate passing a satisfactory examination in the various departments taught, after having attended two courses of medical instruction, the last being taken at this institution.

Such an institution, conducted in the mode in which this was managed before its incorporation, will soon become one of which its enterprising originators may well be proud, and which will be of vast importance to the cause of medical education. The utter futility of making the improvements in the mode of instruction in our medical colleges, which are desired by some prominent members of the profession, must be apparent to every one who has seen much of medical teaching. That students would learn more, and be better prepared to practice their profession if colleges had longer sessions, more demonstration in the teachings of their professors, and less crowding of important facts with no illustration, there can be no doubt; but in our opinion—and we think the proceedings of the late convention of teachers at Louisville indicate a similar view taken by some of our most distinguished professors—this is not now practicable. It may be that the time has not yet arrived, but we are certain that such an extensive modification of the course of medical instruction cannot be generally adopted. Such an experiment is now being attempted in Chicago by the Lind University; but, with all due respect for the persons engaged in the undertaking, and with an admiration for the motives which are at the bottom of it, we think that the school will be unsuccessful. We have seldom heard a remark more true than one which was lately made to us by an eminent Southern teacher connected with a flourishing summer school, which was, "that ordinary office instruction was generally perfectly useless." Physicians seldom examine their students; abstract treatises are put into their hands to read, with no application or illustration of their contents by practice; and frequently, in the practical departments, works are read by students, as text-books, which are years behind the age. We venture to assert that three-fourths of the medical students who attend lectures in this country have passed the period of their office instruction in endeavoring to learn anatomy without dissection, chemistry without any definite idea of the simplest combination, physiology from an old edition of Carpenter, perhaps, and the practical branches as they existed a quarter of a century ago.

This deficiency in the preparatory education of students, and in their mode of study between the lecture terms, cannot yet be supplied by our medical colleges; but it can be and is made up by preparatory schools, conducted by ambitious young men who are conversant with the present state of science, and experienced in imparting instruction. We rejoice, then, to see the incorporation of the New York Preparatory School, where the winter's instruction in the several colleges can be fixed in the mind, and studies pursued advantageously in the intervals between the sessions. The vast resources of the city of New York present unequaled advantages for all kinds of medical instruction, and we are glad to see, by this announcement, that they are taken advantage of. A suitable building has already been erected on Thirteenth-street, near Fourth-avenue. We wish the enterprise the success which it merits, and which, we think, it is sure to attain.

